# Supportive Care for People With Disabilities as Working Partnerships With Their Assistance Dogs Are Ending: A Perspective From Veterinary Oncology

**DOI:** 10.3389/fvets.2019.00309

**Published:** 2019-09-25

**Authors:** Alice E. Villalobos

**Affiliations:** Pawspice and Animal Oncology Consultation Service, Woodland Hills, CA, United States

**Keywords:** disability, euthanasia, veterinary oncology, quality of life, RETHINK, good shepherd, self-care

## Abstract

People with disabilities and those working to train, provide and support assistance animals, along with their veterinary teams, would all benefit if they RETHINK their perspective and viewpoint, and roles when these very special relationships come to an end. The end of the relationship may be when the assistance animal must retire, must be redirected, or euthanized due to illness or cancer. The loss or separation at the end of an assistance animal's service marks a heavy loss for the disabled person. Emotions emerge when the assistance animal is sick or has developed cancer or is approaching the difficult period known as “end of life.” Anticipatory grief and heartbreak may be very difficult to manage and support. We can help ease the burden of decision making when euthanasia is needed for the assistance animal. If the disabled person takes on the good shepherd role and if the veterinary team emulates the minister or Mother Nature's role at the end of life or at the end of the working relationship, heartache may be lifted from both sides of the leash.

## Introduction

The importance of the special relationship between the veterinarian and a disabled handler-service dog team escalates when the dog's job becomes jeopardized due to illness, aging, or behavior challenges. The strong human-animal bond shared between handlers and their assistance animals involves emotional, physical, and spiritual dependency. A threatened bond resembles dealing with the loss of a spouse, or person that provides complete support. Initially a close working relationship sensitizes the veterinary team to the special needs of the person with disabilities, setting the stage for addressing difficult situations. While the assistance animal is still working, the veterinarian can guide the handler in fulfilling the caregiving role, and regularly monitoring wellness. If the dog declines, the veterinarian can counsel, and offer options. When deciding to retire the dog or provide euthanasia, expertise at this heartbreaking moment is crucial. The veterinary team's relationship with the person and dog merits special consideration to assure effective, convenient and supportive care for both, including:

1. Building a close working relationship from the beginning.
2. Guiding the client in assessing the dog's quality of life, wellness, and providing palliative care when needed.
3. Facilitating the client in making difficult decisions, preparing for changes, and taking necessary next steps.
4. Assisting the client during separation from the working relationship.
5. Supporting the family facing difficult decisions.
6. Supporting the handler in accepting their loss via euthanasia or ending the working relationship.

People with disabilities and their assistance animals, along with their veterinary teams, can all benefit by conjointly rethinking their perspective, and viewpoints when this special working relationship ends. The assistance animal may need to retire, be redirected, or may be euthanized due to severe illness, or terminal cancer. This loss or separation marks a heavy loss for the handler with disabilities who views the dog as an essential lifeline (1). Strong emotions, such as sorrow and anticipatory grief, may emerge when the assistance animal has developed cancer or is approaching the period known as “end of life.” Easing the burden of decision making about euthanasia is very important. If the veterinary team emulates the minister role or the role of Mother Nature's helping hand; and if the disabled person emulates the good shepherd role, when the assistance animal's working relationship or life is ending, heartache can be alleviated on both sides of the working harness (2).

## An Assistance Dog with Cancer: Difficult Decisions

Lucy, an 11 year old female, neutered Golden Retriever was an assistance dog for a very spirited person who was wheel-chair bound since childhood. Lucy developed a mast cell tumor near her left knee, which enlarged during the month before being surgically removed.

The biopsy reported a low-grade mast cell tumor, but residual cancer cells remained at the surgical site. The mitotic index (rate of cell division) score of 5 was higher than a low-grade score of below 4. The standard options to manage Lucy's cancer were: more surgery, radiation therapy, and chemotherapy; all were declined by Lucy's handler and her mother. At the first post op consultation, Lucy's local lymph node, and other examinations for metastatic cancer cells were negative. Therefore, a new ablative technology was offered for Lucy: electrochemotherapy (ECT) or electroporation (EP), which kills residual cancer cells at the surgical site without surgery (3). Lucy began working again soon after her EP.

After 8 months, Lucy's cancer spread, causing severe symptoms. Lucy began an end of life Pawspice program, providing gentle palliative cancer care, while also alleviating pain, and other distressful symptoms.

The treatment goal was to restore and maintain Lucy's quality of life. Lucy's handler declined standard IV chemotherapy but authorized the use of steroids and oral chemotherapy. Lucy responded quickly to treatment and worked again for 6 additional weeks. The family was counseled that with every day, Lucy was giving them a very sweet and prolonged farewell.

## Contrasts between Wild and Domesticated Animals

During end-of-life consultations, clients can be encouraged to consider adopting this realistic philosophy. When animals became domesticated, they no longer could separate from their pack, when it was their time to die. Wild animals in decline fall behind their pack or they may separate themselves from the pack and lay under a bush to wait for death from harsh elements, or predation. A prolonged, lingering phase at the end of life is rare for weak, or sick animals in natural habitats. The natural laws of predator-prey mean that frail animals in the wilderness do not linger for long: sick and debilitated animals cannot keep up their daily routine for survival. Unprotected, they are subject to the harshness of Mother Nature's quick hand due to the elements that cause dehydration, cold or heat; and they become prey, entering the food cycle.

Humans domesticated animals and adopted the ancient contract of the good shepherd to care for them, including helping to separate pets at end-of-life when their quality of life declines to a low level, or if they suffer relentlessly. We assume the responsibility to help them depart with a compassionate death. We help provide our assistance animals with the gift of a loving bond-centered euthanasia, assuring that they will have a peaceful and painless passage as we escort them through their transition.

Handlers with disabilities can learn that their assistance animals totally rely on them, as their good shepherds. They can help their assistance animals make a peaceful transition at end of life. If their assistance animals were in the wild, at the end of life, they would have separated themselves from their pack to go off under a bush to await their death. Disabled clients and their families can be counseled that when their loyal assistance dogs are terminally ill, they deserve the benefits of a bond-centered euthanasia. This is a final loving gift that assures a peaceful end-of-life transition.

Domesticated dogs, cats, horses and pocket pets depend on our kindness and wisdom to help them transition when it is their time. People with disabilities can redirect their thinking and grief into the noble thought that they are keeping their obligations as the good shepherd, bound by the ancient contract (4). By providing a compassionate euthanasia, that reflects the handler's cultural, religious and social perspectives, the angst over decision making, grief and mourning may be less emotionally painful for disabled handlers as they part with their beloved assistance animals.

When an assistance dog must retire or be retrained for another role, the loss of the relationship can be very distressful and heartbreaking and may require further professional counseling. Maintaining contact with the animal can be comforting by helping the handler know that their former dog is doing well (5, 6).

## Professional Caregivers Positively Rethinking Their Roles

Veterinarians, and those who care for assistance animals, would benefit it they can rethink what a meaningful and spiritual honor it truly is to oversee compassionate euthanasia services, in accordance with the cultural, religious, and social background of the handlers. They can assume the minister role, or the role as Mother Nature's helping hand, for these very important assistance animals, their disabled handlers, and their families.

Those involved with decision making conversations must avoid and help their clients avoid using negative words and phrases such as: kill, take a life, put down, put to sleep, playing God, blue juice, executioner, etc. Such terms contribute to the veterinary staff's depression, ethics fatigue, and compassion fatigue. Instead, use positive words and phrases, such as: help, escort, assist, transition, transformation, lift, Rainbow Bridge, give back, last kindness, merciful, final loving gift, giving them wings, peaceful, and painless passing, etc.

### Self-Care

All veterinarians and their teams need to revise and rethink their perspective of consistently feeling broken hearted and diminished after providing compassionate euthanasia for their beloved patients, especially for assistance animals. Modifying this thinking will lift the spirits of the entire veterinary team.

Veterinary staff involved with sick or terminal assistance animals need to rethink their roles as helping to provide a bond-centered euthanasia. The team's role in the euthanasia process can be framed as a privilege that is parallel to a minister conducting a sacred sacrament or parallel to Mother Nature's ultimate plan. Rather than being a dreaded weight on well-being, compassionate euthanasia can be considered a last rite ceremony. This “sacrament” is empathetically and professionally delivered by the veterinary “clergy” to assure that the assistance animal has a peaceful and painless passage.

### Teaching Staff Members to Be Supportive

Veterinarians can teach their staff how to communicate about euthanasia, so they learn communication skills for providing emotional support by role-playing and practicing the right words to say. The staff's compassionate emotional support for people with disabilities who face loss, whether to early retirement or to euthanasia, is extremely valuable, and a much needed service. If the assistance animal is sick and is being euthanized, the staff can support the person in knowing and assuring them that they are doing the right thing by validating their difficult decision.

### Teaching Handlers With Disabilities Quality of Life Assessment for Their Assistance Dogs

The veterinary team provides medical care to patients during all the stages of their lives. At the end-of-life stage, the team assists in decision making, and provides palliative care to alleviate pain and distress. When treatment is ineffective or futile, society expects veterinarians to spare their patients relentless, and unnecessary suffering by professionally providing a peaceful, and painless passage with the gift of euthanasia. Veterinary teams can feel reverent and honored to serve persons with disabilities at this sensitive time.

The H5M2 (HHHHHMM) Quality of Life Scale for dogs and cats teaches clients to assess criteria for quality of life (7, 8). The scale assists carers in improving and understanding the quality of life of their animals, guiding their decision making, and is available for free download at: www.pawspice.com click: Menu, Library.

**Table:** 

**Quality of Life Scale**	
**H5M2 (HHHHHMM QoL Scale)**	
Caregivers can use this Quality of Life Scale to assess animals and guide decision making for Pawspice care. Use numbers from 0 to 10 (10 is ideal or normal) to score the patient's condition.	
**Score**	**Criterion**
**0–10**	**HURT**—Adequate pain control & breathing ability is top priority. Trouble breathing outweighs all concerns. Is pain being treated properly or not? Can the animal breathe properly? Is supplemental oxygen necessary?
**0–10**	**HUNGER**—Is the pet eating enough? Does hand feeding help? Does the patient need a feeding tube?
**0–10**	**HYDRATION**—Is the pet dehydrated? For patients not drinking enough water, use subcutaneous fluids daily or twice daily to supplement fluid intake.
**0–10**	**HYGIENE**—The pet should be brushed and cleaned, particularly after eliminations. Avoid pressure sores with soft bedding and keep all wounds clean.
**0–10**	**HAPPINESS**—Does the pet express joy and interest? Is the pet responsive to family, toys, etc.? Is the pet depressed, lonely, anxious, bored, or afraid? Can the pet's bed be moved to be close to family activities?
**0–10**	**MOBILITY**—Can the pet get up without assistance? Does the pet need human or mechanical help? Is the dog willing/able to go out for short walks? Is the pet having seizures or stumbling? Some feel euthanasia is preferable to amputation. But a companion animal with 3 legs or limited mobility can be alert, happy and have a very good QoL only if the family is committed to helping their companion animal get around with: ramps, cart, harness, braces, rehab, acupuncture, etc.
**0–10**	**MORE GOOD DAYS THAN BAD**—When bad days outnumber good days, QoL may be too compromised. When a healthy human-animal bond is no longer possible, the family must be made aware that the end is near. The decision for euthanasia needs to be made if the animal has pointless suffering. If death comes peacefully and painlessly at home, that is okay.
*TOTAL	*A total over 35 points represents acceptable life quality to continue with Pawspice/hospice.

*Oncology Outlook, by Dr. Alice Villalobos, Quality of Life Scale Helps Make Final Call, VPN, 09/2004. Adapted for author's textbook, Canine and Feline Geriatric Oncology: Honoring the Human-Animal Bond, Blackwell Publishing, 2007 & 2018, CB, VCNA, IVAPM Palliative Care & Hospice Statement, 2011, Merial's Pre-EVCONC-World Vet Cancer Congress-Round Table, 2012, with permission of Dr. Villalobos & Wiley-Blackwell Publishing, Hoboken, NY*.

In Lucy's case, her handler, along with her mother, used the Quality of Life scale to evaluate Lucy when her mast cell cancer relapsed with recurring symptoms. Lucy's handler and her mother chose compassionate euthanasia for Lucy when her Quality of Life Score dropped below 35. The New York Times featured this Quality of Life Scale in an article titled, “Is it Time? Making End of Life Decisions for Pets,” on 3-13-2019, https://nyti.ms/2Fel9kC (9).

### Emulating the Role as Good Shepherd

Because assistance animals are trained to be constant helpers and companions, people with disabilities may tend to overprotect them. They may also feel desperate, wanting to hang on to their assistance dogs, when it is not in their dog's best interest. The veterinary team should counsel, comfort and enlighten heartbroken clients with disabilities and ease their burden of guilt by reminding them of their good shepherd responsibility and that their terminally ill beloved assistance dogs should not be forced to suffer needlessly.

### Emulating the Role as Chaplain or Mother Nature's Helper

To enhance personal resilience and professional endurance, veterinarians and team members *must* rethink their esteemed and powerful role in the euthanasia process, visualizing their role in a more positive light, as resembling an honored and respected minister; a chaplain, rabbi, priest, high priestess or Mother Nature's helping hand. Think this way vs. being the executioner.

Veterinarians can consider the role that chaplains play at end of life, consoling the bereft and providing emotional comfort, assuring grieving clients with disabilities that they are giving the meaningful gift of a quality death with a bond-centered euthanasia. It is a reverent honor to serve the disabled community at this very vulnerable and sensitive time. Euthanasia should be described to clients with its literal meaning, “good death,” along with gentle words such as: “We will escort your beloved (name of the patient) with a peaceful and painless passing.”

### Providing a Bond-Centered Euthanasia

#### Lucy's Decline

When Lucy's quality of life reached a low point, her loving handler and mother wanted to bring her to our veterinary clinic for her final visit. The family wanted Lucy's final farewell to be surrounded by the veterinary team who knew her well. Our staff validated their difficult decision, since it was best to let Lucy go before she suffered in futility. Our team offered hugs and supported their decision so that Lucy's handler and her mother would not later feel guilty about helping Lucy transition. We assured them that they acted as the good shepherd and that they helped Lucy avoid enduring unnecessary and futile suffering if they had waited much longer.

### Compassionate Planning for Euthanasia

Providing a compassionate euthanasia occasion (with candles, flowers, and poems based on client preferences) should be considered a special personal and professional honor, not a dreaded task. Euthanasia can be viewed as a “balloon” or a “lifter”: an opportunity to kindly oversee and alleviate a heartbreaking event. This process teaches assistance animal care providers to feel better about themselves as they ease the loss for the carers.

### Euthanasia: Recommendations for the Veterinary Team

Many carers prefer home euthanasia as a more comfortable setting for their final farewell; this option should be encouraged and can be facilitated by referral to a house call practice that provides hospice and home euthanasia services.

The exam room setting for compassionate bond-centered euthanasia can be provided as described below (10):

Light candles, bring in flowers, and turn down bright lights.

Assure that the family is unified with the decision for retiring their assistance animal or validate them for making the difficult decision for euthanasia.

Explain the two-step procedure to the family to assure a peaceful and painless passage. The first step is to give a sedative by injection, either intramuscular (IM) or subcutaneous (SQ), to allow the patient to fall asleep peacefully in the presence of the family. Once the pet is sedated, the second step is to provide an intra-organ injection that will cause the heart and breathing to stop. Some doctors prefer to place an IV catheter after sedation.

Avoid separating the pet from their family. If preferring to place an intravenous catheter for the final injection, place the catheter after the IM or SQ sedation is in effect, with the family present. Do not break the bond at this time. Explain to the family that some pets will take a reflex breath up to 2–5 min after they are deceased, so the family will know what to expect and not be alarmed.

For the final intra-organ injection, cover the pet with a towel and instruct the family to massage their pet's head and neck. Under the towel, listen for the heart and then administer the euthanasia solution into the heart, kidney, liver, or as an intraperitoneal injection, which takes longer for the heart to stop. Wait peacefully for the last heartbeat. Pronounce the pet's time of death when the heartbeat becomes inaudible. Document this in the chart.

Invite the family to stay with their deceased pet for whatever time they need. Tell them that this special time is their version of a wake. Validate their decision as being right for the given circumstances.

Read poetry such as: “The Rainbow Bridge.”

Family members can be asked to blow out the candle(s) as a symbol of life's end, before they leave. The family generally leaves their deceased pet at the hospital for afterlife arrangements (cremation, aquamation, burial, paw print, pictures). If at home, the deceased may be taken for after life body care by the doctor's staff or by a service.

### Parting With Lucy

Terina Sprague (Lucy's handler) and Sherril Sprague (Terina's mother) gave us verbal and written consent to publish potentially-identifying images and information about Lucy's very special and interesting case. They were grateful for our pet loss counseling and the emotional support provided during Lucy's care and end of life process. Our entire oncology team hugged them and gave them flowers and Lucy souvenirs: including a special Lucy paw print for Terina and a lock of Lucy's fur in a windowed envelope. We gave Terina photos and an autographed poetry book, *Angel Whiskers*, which includes the Rainbow Bridge poem and other poems that I read during the wake (11). Our veterinary team arranged for Lucy to be cremated with her teddy bear and a special picture of Lucy, wheelchair assistance dog, next to Terina during a favorite “Rolling Along” sporting event.

Losing the assistance dog relationship with Lucy was very difficult for Terina and her mother. They memorialized Lucy with tattoos.

### Afterlife Body Care: Assuming a Role as Funeral Director

It is important to provide personal and private assistance when making afterlife arrangements for cremation or burial of a deceased assistance dog. Ask this simple question, “Have you thought about or planned for cremation or burial?” Never ask the horrible question: “How do you want to *dispose* of the body?” After the decision for private or group cremation, then the veterinary team can assist the client in “making arrangements” with the afterlife service provider. Some useful gestures may include:

Send a sympathy card with your personal notation(s).

Donate to a special cause in the name of the deceased.

Contact the family 1 or 2 days later and ask:

“How is your heart and soul doing?”

Offer additional emotional support and recommend pet loss counseling, with contact information, or a chaplain to help process their grief. Suggest pet loss books such as *So Easy to Love, So Hard to Lose* (12). Recommend a pet loss chat room provided by the Association of Pet Loss and Bereavement at: www.aplb.org.

If children are part of the family, be sure to address their grief.

Instruct the family to set up a shrine with pictures of the pet and to light a candle to honor their human-animal bond.


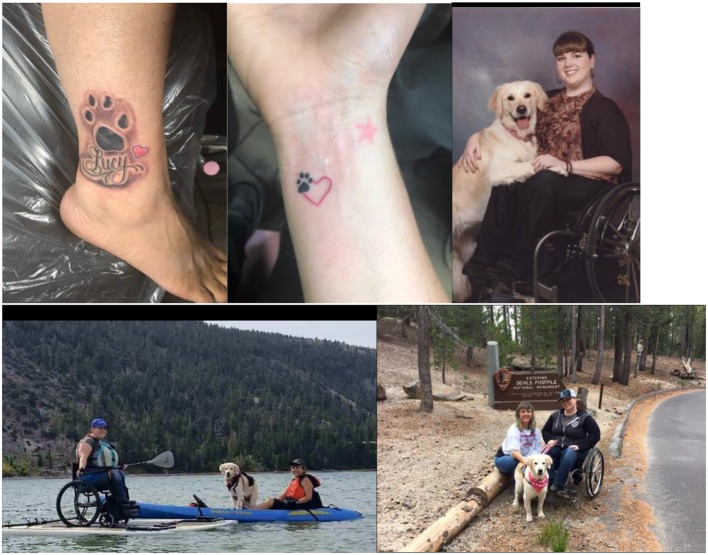


## Summary

As relationships of handlers and their assistance dogs are ending, occupational pressures for the veterinary team may cause stress, depression, compassion fatigue, and ethics fatigue. Rethinking compassionate euthanasia with the good shepherd and minister philosophy can lessen heartbreak and negativity surrounding end of life, as shown in Lucy's case. By elevating veterinarians to emulate the chaplain role and enlightening clients to assume the good shepherd role, we can all be honored escorts for our beloved assistance dogs as they make job transitions or as they transition at end-of-life. Thus, we elevate the spirit and reverence for the very special love within this unique human-animal bond. Please refer to the Resources section at the end of this article for more information about end of life care for animals and emotional support for those providing home care and medical care.

## Author Contributions

The author confirms being the sole contributor of this work and has approved it for publication.

### Conflict of Interest Statement

The author declares that the research was conducted in the absence of any commercial or financial relationships that could be construed as a potential conflict of interest.
